# Retrospective Study on *Staphylococcus aureus* Resistance Profile and Antibiotic Use in a Pediatric Population

**DOI:** 10.3390/antibiotics12091378

**Published:** 2023-08-28

**Authors:** Danilo Buonsenso, Martina Giaimo, Davide Pata, Alessia Rizzi, Barbara Fiori, Teresa Spanu, Antonio Ruggiero, Giorgio Attinà, Marco Piastra, Orazio Genovese, Giovanni Vento, Simonetta Costa, Eloisa Tiberi, Maurizio Sanguinetti, Piero Valentini

**Affiliations:** 1 Department of Woman and Child Health and Public Health, Fondazione Policlinico Universitario A. Gemelli IRCCS, 00168 Rome, Italy; martinagiaimo@virgilio.it (M.G.); davide.pata01@gmail.com (D.P.); antonio.ruggiero@policlinicogemelli.it (A.R.); giorgio.attina@policlinicogemelli.it (G.A.); giovanni.vento@unicatt.it (G.V.); simonetta.costa@policlinicogemelli.it (S.C.); eloisa.tiberi@policlinicogemelli.it (E.T.); piero.valentini@unicatt.it (P.V.); 2 Global Health Center, Università Cattolica del Sacro Cuore, 00168 Rome, Italy; 3 Medicine and Surgery, Università Cattolica del Sacro Cuore, 00168 Rome, Italy; alessia.rizzi01@icatt.it; 4 Department of Laboratory Sciences and Infectious Disease, Fondazione Policlinico Universitario A. Gemelli IRCCS, 00168 Rome, Italy; barbara.fiori@policlinicogemelli.it (B.F.); teresa.spanu@policlinicogemelli.it (T.S.); maurizio.sanguinetti@unicatt.it (M.S.); 5 Department of Emergency, Anesthesiological and Resuscitation Sciences, Fondazione Policlinico Universitario A. Gemelli IRCCS, 00168 Rome, Italy; marco.piastra@unicatt.it (M.P.); orazio.genovese@policlinicogemelli.it (O.G.)

**Keywords:** *Staphylococcus aureus*, antibiotics, antibiotic resistance

## Abstract

The growing phenomenon of antibiotic resistance and the presence of limited data concerning the pediatric area prompted us to focus on *Staphylococcus aureus* infection in this study, its antibiotic resistance profile, and the therapeutic management of affected children. We conducted a retrospective study by collecting clinical data on infants and children with antibiogram-associated *S. aureus* infection. We enrolled 1210 patients with a mean age of 0.9 years. We analyzed the resistance patterns and found 61.5% resistance to oxacillin, 58.4% resistance to cephalosporins, 41.6% resistance to aminoglycosides, and 38.3% resistance to fluoroquinolones. Importantly, we found no resistance to glycopeptides, a key antibiotic for *MRSA* infections whose resistance is increasing worldwide. We also found that the main risk factors associated with antibiotic resistance are being aged between 0 and 28 days, the presence of devices, and comorbidities. Antibiotic resistance is a growing concern; knowing the resistance profiles makes it possible to better target the therapy; however, it is important to use antibiotics according to the principles of antibiotic stewardship to limit their spread.

## 1. Introduction

*Staphylococcus aureus* is one of the most important human pathogens whose concern can range from mild skin and soft tissue infections to life-threatening bacteremia, and it contributes to both community-associated and healthcare-associated infections, with a high clinical burden for infants worldwide [1]. *S. aureus* colonizes approximately 30% of the human population asymptomatically in the nostrils, either transiently or persistently; therefore, it can be considered a human commensal [2]. As *S. aureus* colonization is a risk factor for infection, preventive strategies aim to optimize hygiene measures and decolonization regimes for outpatients and critically ill children with prolonged hospitalization [3]. Transmission occurs following contact with a person or infected surfaces and, in some cases, after ingestion of contaminated food. Open wounds on the skin, basic diseases, the presence of implantable devices, and long hospital stays are the main risk factors [4]. Nosocomial infections related to the carriage of *S. aureus* are rare but serious. Mechanical ventilation, surgery during pediatric intensive care unit stay, and anemia are factors associated with healthcare-associated infections [5]. Skin infection with epidermolytic toxin-producing staphylococci is the cause of acute epidermal necrolysis (Ritter’s disease in infants or Lyell’s disease in young children) [6]. Bacteremia is usually complicated by metastatic abscesses, which are peculiar elements of invasive inflammatory lesions of *S. aureus* [7], with different localizations that can reach the skeletal system, causing the destruction of bone tissue and septic arthritis [8]. In patients with underlying or recent lung disease, this bacterium can lead to the formation of abscesses and lung consolidation. In the most severe cases, there may be an onset of toxic shock, a clinical syndrome characterized by multi-system intoxication with the onset of fever, hypotension, and diffuse erythematous macular rash with high mortality in the absence of timely antibiotic therapy and elimination of the source of infection [9]. In a prospective, cross-sectional study of children admitted to hospitals in Australia and New Zealand, a high incidence of *S. aureus* bacteremia was demonstrated, and necrotizing pneumonia and multifocal infection were predictors of mortality, whereas consultation with an infectious disease specialist was protective [10].

*S. aureus* is included in the group of “ESKAPE” bacteria, which comprise the multidrug-resistant pathogens that are currently considered the biggest concern for humanity [1,11]. It is ubiquitous in the environment, and it shows remarkable resistance to unfavorable environmental conditions, which is one of the reasons for its important pathogenicity and acquisition of antibiotic resistance [7,11]. The development of an effective vaccine against *S. aureus* has been a major goal in recent decades, but despite the wealth of understanding of its pathogenesis, it has failed in all studies [12].

Bacterial resistance to antibiotics, particularly multiple antibiotics, has become a serious health emergency worldwide [13]. According to the WHO, 700,000 people around the world die every year from infections caused by antibiotic-resistant bacteria. There is a definite relationship between the excessive and inappropriate consumption of antibiotics and the emergence of resistance phenomenon, which is present in both humans and animals [14,15].

Acquired resistance is present in certain strains of the same species or genus. In some cases, it can be very frequent, e.g., penicillinase production in staphylococci is present in more than 90% of strains. Antibiotic resistance in *S. aureus* is associated with the presence of Mobile Genetic Elements, the primary means by which genetic information is exchanged between bacteria via horizontal gene transfer, which plays a key role in the ability of *S. aureus* to adapt to environmental stresses, including exposure to antibiotics [16,17]. *S. aureus* strains in general contain a relatively large variety of Mobile Genetic Elements, including plasmids, transposons, bacteriophages, pathogenicity islands, and staphylococcal cassette chromosomes (SCC) [18].

The virulence and rapid transmission of methicillin-resistant *S. aureus* infections have raised interest in understanding the pathogenesis of this organism [19]. There are two main types of infections: healthcare-associated (HA)-MRSA and community-associated (CA)-MRSA. Currently, the most widespread *CA-MRSA* strain is among the most common causes of skin and soft tissue infections in emergency departments in the US. Up to 10% of these infections are invasive, such as sepsis, meningitis, osteomyelitis, and necrotizing pneumonia [20]. Several studies have highlighted two possible molecular markers for *CA-MRSA*: the gene for Panton-Valentine leucocidin (PVL), which encodes a toxin that acts as a virulence factor, and SCCmec IV, a horizontally transferred element that confers resistance to methicillin but maintains susceptibility to other antibiotics [21].

A recent multicenter study showed a high prevalence of PVL in *CA-MRSA* infections in children, whereas SCCmec IV was more commonly isolated from *HA-MRSA* isolates [22]. In another study carried out from 2009 to 2015, *S. aureus* isolated from pediatric sterile sites was selected and analyzed using polymerase chain reaction to detect the mecA and PVL genes. The rate of methicillin-resistant *S. aureus* was 37.7%. Skin and soft tissue infections were significantly associated with PVL-positive isolates [23]. In a prospective study of a cohort of pediatric patients from 13 centers in 7 European countries, 85 children were included; PVL-positive isolates were 17%, and 6% of the isolates were *MRSA*. Multivariate analysis identified the presence of PVL as the only factor independently associated with severe outcomes, regardless of methicillin resistance [24]. The current healthcare policy envisages fewer hospitalizations and more use of outpatient centers. This makes it much more difficult to apply traditional definitions to classify infections. In addition, it is known that patients may be colonized by *HA-MRSA* for years before developing an infection, and cases of nosocomial outbreaks of *CA-MRSA* have been reported [11], so any attempt to distinguish *CA-MRSA* from *HA-MRSA* using purely epidemiological criteria cannot be considered sensitive or specific [25,26].

Antimicrobial stewardship is defined as the careful and responsible management of antimicrobial use. With rising antimicrobial resistance rates worldwide and the development of very few new antibiotics, existing antibiotics are becoming a limited resource. Antibiotics should be used for the shortest time possible, using molecules with the narrowest spectrum of action. Empirical treatment should be tailored to the type of infection and resistance profile present in each country or region [27,28].

The limited international literature on antibiotic resistance in pediatric patients prompted us to carry out a retrospective study on children admitted to the pediatric wards of our University Hospital in Rome, over the last few years, focusing on patterns of *S. aureus* antimicrobial resistance, antibiotic use, and risk factors for *S. aureus* resistant species in children.

## 2. Materials and Methods

### 2.1. Study Design and Participants

A retrospective study was carried out evaluating the medical records of pediatric patients admitted to the departments of Pediatrics, Neonatology with Neonatal Intensive Care Unit, Child Neuropsychiatry, Pediatric Emergency Department, Pediatric Intensive Care Unit and Child Neurosurgery of IRCCS Policlinico A. Gemelli (Università Cattolica del Sacro Cuore), Rome, Italy. The records studied concerned admissions made between 11 February 2016 and 30 October 2020 that, among the diagnoses at discharge, reported *S. aureus* infection.

### 2.2. Inclusion Criteria

All patients in whom one of the biological samples was positive for *S. aureus* were included in the study, and the resistance rate towards the most used antibiotics was analyzed. Patients over 18 years of age, as well as patients who did not present an antibiogram in the file, were excluded.

### 2.3. Collected Variables and Microbiological Studies

The demographic and clinical characteristics studied included gender, age at onset, reason for admission, total days of hospitalization, admission to the neonatal or pediatric intensive care unit, number of visits to the emergency department, presence of comorbidities, and use of devices (tracheotomy, SNG, and catheters). Antibiotic therapies administered during hospitalization were also assessed. The outcome measures were patterns of resistance and factors associated with pediatric age.

Species identification was performed by matrix-assisted laser desorption/ionization time-of-flight (MALDI) mass spectrometry using the Bruker Daltonics MALDI Biotyper system (Bruker Daltonics, Bremen, Germany). The antibiotic susceptibility profiles of the *S. aureus* isolates were determined using the VITEK 2 system (bioMérieux, Rome, Italy), following the manufacturer’s instructions. The antimicrobial sensitivity breakpoints were interpreted according to the current EUCAST breakpoint for *S. aureus* [29].

### 2.4. Primary and Secondary Aims

The primary aim of this study was to describe the main patterns of antibiotic resistance in children with *S. aureus* infections.

Secondarily, the analysis focused on the possible antibiotic therapies that could be prescribed in patients infected by *S. aureus* in relation to the antibiotic sensitivities revealed by the antibiogram; the study also evaluated the association between the possible factors of increased susceptibility such as the presence of comorbidities, admission to intensive care, days of hospitalization, emergency room accesses, use of devices, and the prevalence of resistance. Finally, by analyzing the therapies administered and antibiotic susceptibilities, we sought to identify the optimal management of patients, with particular emphasis on the possibility of changing therapy.

### 2.5. Statistical Analyses

The following analysis was carried out:

- A descriptive statistical analysis: calculated median and interquartile range for numeric variables and absolute and percentage frequencies for categorical variables;
- An inferential statistical analysis;
- Univariate analysis for comparison of the frequency of resistance to the investigated antibiotics between categories of categorical variables using the χ^2^ test (or Fisher’s exact test in the case of expected absolute frequencies in at least one of the cells of the contingency table less than 5);
- Non-parametric Mann–Whitney test for the comparison of numerical variables with non-normal distribution between the investigated antibiotic-resistant and non-resistant groups;
- Multivariate analysis using logistic regression for the calculation of the odds ratio and relative 95% confidence interval.

The analyses were performed using the statistical package STATA version 16.1, and the graphs were constructed using Excel.

The study was approved by the Ethics committee of our hospital (ID 5741, n. 0015790/23).

## 3. Results

### 3.1. Study Population

A total of 1210 children were included in this study, of which 702 (58.0%) were male, and 508 (42.0%) were female. The mean age at diagnosis was 0.9 years with a range between 0 and 18 years (Table 1).

The patients were divided into three categories according to age:

- Newborns (0–28 days): 484 patients (40%);
- Infants (29–90 days): 170 patients (14%);
- Children (older than 90 days): 556 patients (46%).

There were 560 patients admitted to the neonatal intensive care unit (46.2%) and 219 patients admitted to the pediatric intensive care unit (18.1%).

In patients presenting with a positive finding for *S. aureus*, the isolation sites were as follows (more information in Table 2)

- Pharyngeal swab in 46% of cases;
- Bronchoalveolar lavage (BAL) in 35% of cases;
- Central venous catheter (CVC) in 13% of cases;
- Urine in 4% of cases;
- Other sites in 2% of cases.

The analysis of possible susceptibility factors indicated that there were 830 patients with comorbidities (68.6%), divided into the following categories: hematological (23.7%), gastrointestinal (19.4%), infectious (23.1%), renal (18.9%), and other causes (14.9%).

Devices, such as central venous catheter, tracheostomy, urinary catheter, percutaneous endoscopic gastrostomy (PEG), and arterial catheter, were present in 814 patients (67.3%).

### 3.2. Pattern of Antibiotic Resistance in S. aureus Isolates

**Figure 1:**
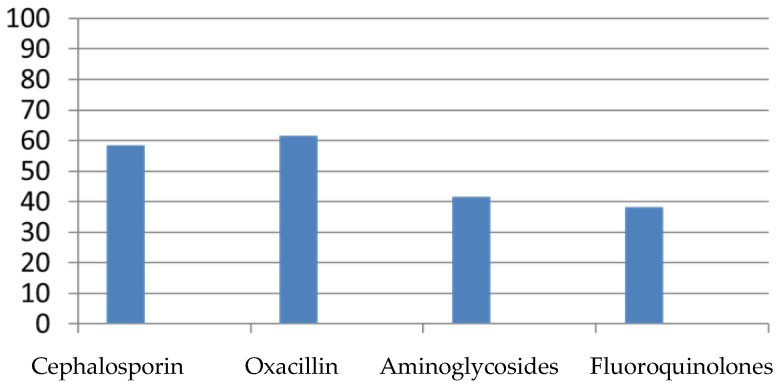
Antibiotic resistance to the main antibiotics was analyzed.

Analyzing antibiotic resistance in the entire test population was stratified as shown in Figure 1:

Oxacillin-resistant (Table 3) patients accounted for 61.5% (744 patients). In total, 57.7% (699 patients) were treated empirically with oxacillin. Patients treated with oxacillin admitted to the ICU accounted for 19% (132 patients), particularly 12% in the neonatal ICU (84 patients) and 7% in the pediatric ICU (49 patients). Of these cases, 22% (154 patients) had comorbidities, 34% (237 patients) were device carriers, 53% (370 patients) were resistant to the empirical antibiotic used, and 27% (189 patients) had undergone modification of antibiotic therapy, taking a second-line antibiotic. Age between 0 and 28 days (*p* < 0.001) and the presence of devices (*p* 0.001) were significantly associated with the isolation of oxacillin-resistant *S. aureus*.

The isolation sites of oxacillin-resistant bacteria were as follows:

- Pharyngeal swab in 24.6% of cases (298 patients);
- Broncho-alveolar lavage (BAL) in 8.6% of cases (104 patients);
- Central venous catheter (CVC) in 0.5% of cases (6 patients);
- Urine in 1.32% of cases (16 patients);
- Other sites in 0.6% of cases (8 patients).

Cephalosporin-resistant (Table 4) patients accounted for 58.4% (706 patients). In total, 10.6% (128 patients) were treated with cephalosporins. Patients treated with cephalosporins admitted to the ICU accounted for 13% (17 patients), particularly 6% in the neonatal ICU (8 patients) and 7% in the pediatric ICU (9 patients). Of these cases, 28% (36 patients) had comorbidities, 37% (47 patients) were device carriers, 49% (63 patients) were resistant to the antibiotic used, and 29% (37 patients) changed antibiotic therapy, taking a second-line antibiotic. None of the patients showed a therapeutic downshift. An age range of 0–28 days (*p* < 0.001), the presence of comorbidities (*p* 0.02), and the presence of devices (*p* < 0.001) were significantly associated with the isolation of cephalosporin-resistant *S. aureus*. The isolation sites of cephalosporin-resistant bacteria were as follows:

- Pharyngeal swab in 15.2% of cases (184 patients);
- Broncho-alveolar lavage (BAL) in 16.4% of cases (199 patients);
- Central venous catheter (CVC) in 0.4% of cases (5 patients);
- Urine in 0.6% of cases (8 patients);
- Other sites in 0.5% of cases (6 patients).

Patients resistant to aminoglycosides (Table 5) accounted for 41.6% (503 patients). A total of 51.5% (623 patients) were treated with these antibiotics as an empirical therapy. Patients treated with aminoglycosides admitted to the intensive care unit accounted for 64% (399 patients), particularly 52% in the neonatal intensive care unit (324 patients) and 12% in the pediatric intensive care unit (75 patients). In total, 23% (143 patients) had comorbidities; 65% (405 patients) were device carriers, 33% (206 patients) were resistant to the antibiotic used, and 19% (118 patients) changed antibiotic therapy, taking a second-line antibiotic. None of the patients showed a therapeutic downshift. Age between 0 and 28 days (*p* < 0.03), previous admission to an intensive care unit (*p* < 0.01), and the presence of comorbidities (*p* < 0.002) were significantly associated with the isolation of aminoglycoside-resistant *S. aureus*. The isolation sites of aminoglycosides-resistant bacteria were as follows:

- Pharyngeal swab in 3.2% of cases (39 patients);
- Broncho-alveolar lavage (BAL) in 5.6% of cases (68 patients);
- Central venous catheter (CVC) in 7.2% of cases (87 patients);
- Urine in 1.2% of cases (13 patients);
- Other sites in 0.57% of cases (7 patients).

Patients resistant to fluoroquinolones (Table 6) accounted for 38.18% (461 patients). In total, 0.9% (11 patients) were treated with fluoroquinolones. Patients treated with fluoroquinolones admitted to the ICU accounted for 27% (3 patients), particularly 9% in the neonatal ICU (1 patient) and 18% in the pediatric ICU (2 patients). Of the cases, 29% (3 patients) had comorbidities, 27% (3 patients) were device wearers, 55% (6 patients) were resistant to the antibiotic used, and 45% (5 patients) changed antibiotic therapy, taking a third-line antibiotic. None of the patients showed a therapeutic downshift. The presence of devices (*p* < 0.001) and the presence of comorbidities, especially hematological (*p* < 0.01), were significantly associated with the isolation of fluoroquinolone-resistant *S. aureus*. The isolation sites in fluoroquinolones-resistant patients were as follows:

- Pharyngeal swab in 2.3% of cases (36 patients);
- Broncho-alveolar lavage (BAL) in 4.4% of cases (53 patients);
- Central venous catheter (CVC) in 4.9% of cases (59 patients);
- Urine in 0.91% of cases (11 patients);
- Other sites in 0.25% of cases (3 patients).

None of the *S. aureus* strains were resistant to glycopeptides. A total of 4.8% (58 patients) received glycopeptide therapy after the antibiogram results: in these cases, the *S. aureus* strains were resistant to oxacillin, amoxicillin/clavulanate, aminoglycosides, cephalosporins, and fluoroquinolones.

### 3.3. Empiric and Targeted Antibiotics Used

Following the resistance analysis, the differences in treatment before and after the antibiogram results were evaluated.

The treatment of the 1210 patients under review was as follows:

- Oxacillin or β-lactam therapy was initiated in 48% of cases;
- 42% began therapy with aminoglycosides;
- Cephalosporin therapy was initiated in 9% of cases;
- Fluoroquinolones were initiated in 1% of cases;
- No patients began therapy with glycopeptides.

**Figure 2:**
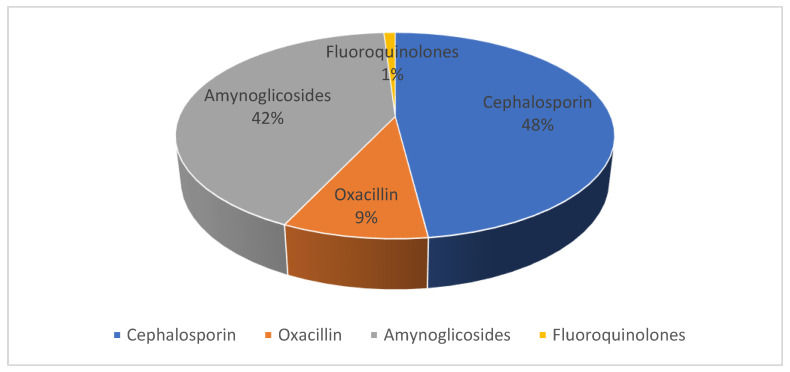
Empiric antibiotic therapy before antibiogram results.

**Figure 3:**
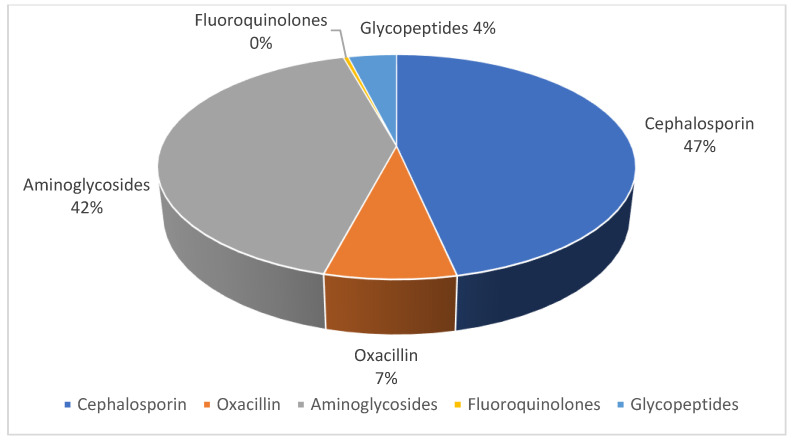
Antibiotic therapy after antibiogram results.

The change in therapy was then assessed following the results of the antibiogram, in which an increase in the administration of glycopeptides can be observed (3.9%) in Figure 2 and Figure 3.

## 4. Discussion

In this study, we evaluated the antibiotic resistance patterns of *S. aureus* and antibiotic use in a large cohort of pediatric patients. Overall, it was found that the oxacillin resistance rate was 61.5%, followed by the cephalosporin resistance rate (58.4%). The resistance rates to aminoglycosides and fluoroquinolones were 41.6% and 38.18%, respectively. None of the isolates was resistant to glycopeptides. This last point is a particularly important observation since the rate of resistance to glycopeptides by *S. aureus* is progressively increasing, as demonstrated in the literature by systematic reviews that show a prevalence of glycopeptide-resistant *S. aureus* strains from 4.68% (1997) to 7.93% (2017) [17]. Vancomycin, a glycopeptide antibiotic that inhibits cell wall biosynthesis, remains the drug of choice for the treatment of severe *MRSA* infections. *S. aureus* strains exhibiting increased resistance to vancomycin, known as vancomycin intermediate-resistant *S. aureus* (*VISA*), were discovered in the 1990s. *S. aureus* isolates with complete resistance to vancomycin are termed vancomycin-resistant *S. aureus* (*VRSA),* and they were first reported in the US in 2002. Although the treatment of *VRSA* infections is challenging, the total number of human *VRSA* infections reported to date is limited. In comparison, the burden of *VISA* is relatively high, and it is associated with persistent infections, vancomycin treatment failure, and poor clinical outcomes [18]. In a recent US study, reduced sensitivity to vancomycin was common (72% of cases) and was associated with a longer duration of bacteremia, but not with treatment failure. Treatment failure was more common in *MRSA* infections than in *MSSA* infections. Empirical vancomycin monotherapy increased the likelihood of treatment failure in *MRSA* infections [30]. A surveillance study conducted in 2003 in 15 hospitals in Taiwan showed that the prevalence rates of *VRSA* increased from 0.7% to 2.7% between 2012 and 2013, with a total of 622 vancomycin-resistant isolates. There was also a higher use of second-line antibiotics, such as Ceftazidime and Meropenem, in these hospitals compared to European countries [31]. These data led us to focus more on the criteria for using glycopeptides, given their importance and the clinical impact of an increase in resistant bacterial strains. It is precisely in relation to the increasing number of cases of resistance to treatment with glycopeptides that the use of linezolid may be given more consideration for the treatment of *MRSA* infections [32]. In a study of 321 pediatric patients with *MRSA* infections, the therapeutic efficacy of vancomycin was compared with that of linezolid. Linezolid was associated with higher efficacy than vancomycin for *MRSA*-related infections in terms of clinical treatment success; it is also associated with fewer episodes of nephrotoxicity, and it has the advantage of a convenient oral formulation with 100% bioavailability and lower costs [33]. In a more recent meta-analysis comparing linezolid and vancomycin for the treatment of nosocomial pneumonia in children over 12 years of age, it was found that linezolid was not superior to glycopeptide antibiotics for the endpoints of clinical success, microbiological success, or mortality, and the risk of adverse events was not different between the two classes of antibiotics [34].

When evaluating the changes in therapy before and after the antibiogram was performed, glycopeptide administration was never performed as the first-line therapy. This is reassuring as it is in line with the principles of antibiotic stewardship. However, extra effort should be made regarding the transition from empirical to targeted therapy in light of the antibiogram results; it was found that aminoglycosides and cephalosporins were frequently used as empirical therapy, whereas targeted therapies to avoid these classes of antibiotics were not always established once antibiograms became available. This error in patient management is one of the main causes of the increased resistance in *S. aureus* strains. Possible explanations for this behavior are fear of antibiotic change, habit, and a good response to the antibiotic used. However, it is important to continue implementing programs that tend to reduce the use of expensive broad-spectrum antibiotics to combat antibiotic resistance. After the antibiogram result, we observed an increase in the administration of glycopeptides (3.9%) in patients in whom bacterial resistance occurred, all of whom had risk factors related to the development of bacterial resistance, such as the presence of devices and the presence of comorbidities. Our study shows that, in the case of antibiotic resistance, pediatricians generally adjust therapy by choosing a sensitive drug: indeed, there is an increase in the administration of aminoglycosides (+29%), fluoroquinolones (+28%), and glycopeptides (+4%). Moreover, in 2% of oxacillin-sensitive bacterial infections, cephalosporin was used as therapy, and in 4.1% of cephalosporin-sensitive germ infections, aminoglycoside was used as therapy. In a study conducted in 2016 in Laos, health workers were interviewed and the medical records of children under five years of age were examined. Among the 54 health workers interviewed, 85.2% were aware of the standard treatment guidelines, but only 77.8% adhered to the guidelines. The difference in correct drug administration lay in the location of the workers, showing a lower adherence to guidelines and antimicrobial stewardship in district hospitals due to a greater fear of treatment failure, which is also associated with a longer duration of antibiotic intake [35]. The use of aminoglycosides and cephalosporins as empirical therapy has been carried out mainly in premature patients in order to reduce the risk of a worsening of the clinical picture with the development of sepsis; the use of these drug classes is associated with a 23.7% reduction in sepsis-related mortality rates, as shown by studies conducted in the US, in patients with skin infections and in patients admitted to the intensive care unit [36].

The epidemiology of *S. aureus* infection in children is dynamic. The population-weighted *MRSA* rate in the EU has been declining for many years; like recent trends observed in adults, the rate of pediatric *MRSA* infections also appears to be declining in the US between 2005 and 2014 [37]. However, the ECDC study on the health burden of antimicrobial resistance reported an increase in the estimated incidence of *MRSA* between 2007 and 2015. Further analysis of the age-group-specific incidence as part of the ECDC study found that it is mainly related to infants (in addition to people aged 55 years or above) [38]. Our study population reflects data compiled by the ECDC with an average age of 0.9 years, with 54% of patients under 90 days of age and, therefore, belonging to the group with the highest prevalence of resistance found. With these data, Italy is confirmed as one of the European countries with the highest rate of microbial resistance, together with Portugal, Greece, and Romania [38]. This is mainly due to the ease with which antibiotics can be purchased in these countries directly from community pharmacies without a prescription. A study conducted in Catalonia in 2009 showed that antibiotics were sold without a prescription in 79.7% of the pharmacies studied. In Greece, only 79% of patients reported receiving antibiotics on prescription [39]. A significant decrease in the EU population-weighted percentage of *MRSA* isolates was reported during the period 2017−2021, from 18.4% to 15.8%. Nevertheless, *MRSA* remains an important pathogen in the EU, with high prevalence in several countries. Among laboratories that consistently reported data each year in the period 2017–2021, the decrease in the annual number of *MRSA* isolates reported for the period 2019–2020 reversed to some extent during the period 2020–2021. Specifically, prior to 2020, annual reductions in the proportion of *MRSA* were explained by the relatively large and continuous increase in the number of reported methicillin-susceptible *S. aureus* infections, whereas the annual number of reported *MRSA* infections remained relatively stable [40]. In a recent Spanish study that aimed to discover the rate of *MRSA*-positive cultures in Spanish pediatric emergency departments, the overall *MRSA* rate was one in six staphylococcal infections. Higher rates of *MRSA* were found in specimens of suppurative skin lesions and in children from abroad or with a history of previous *MRSA* infection. The study concluded that early drainage of suppurative skin lesions is essential and switching to an antibiotic with *MRSA* should be considered [41]. In a retrospective observational study of pediatric clinical cultures performed between 2005 and 2017, although methicillin resistance decreased, resistance to clindamycin and trimethoprim-sulfamethoxazole increased significantly, especially among isolates of community origin [42].

In our study, we observed an increased resistance of *S. aureus* to the most used antibiotics. While 61.5% of *S. aureus* strains were resistant to oxacillin, 41.6% and 38.18% of the strains were resistant to aminoglycosides and fluoroquinolones, respectively. Pediatric practice includes different patient groups, both clinically and epidemiologically. Babies and infants differ significantly from each other, older children, and adults. For this reason, our study is particularly significant as it adds important data to the patterns of antibiotic resistance in the pediatric population, as most studies in the literature are conducted on adult populations. The subgroups of the pediatric population differ in the pharmacokinetic aspects of antimicrobial drugs, depending on the age of the patient. Infants have a larger extracellular fluid volume, immature liver and kidney functions, and higher plasma concentrations of bilirubin and non-esterified fatty acids. The water content is higher in premature infants than in term infants; penicillin, cephalosporins, and aminoglycosides are water-soluble and are distributed in a larger volume in premature infants than in term infants. These antibiotics are mainly eliminated by the kidneys, and their renal glomerular filtration and tubular secretion are reduced in infants, so clearance is reduced in infants compared to children. Therefore, in children, the volume of distribution of penicillin, cephalosporins, and aminoglycosides is greater than that in adults due to their higher body water content, so drug administration per kg body weight is required [43].

Of the patients treated with oxacillin, 12% (84 patients) had no risk factors for the development of bacterial resistance (device, intensive care unit admission, and comorbidities), whereas of the patients treated with cephalosporins, 6% (8 patients) had risk factors, so the use of first-line β-lactams was appropriate. Regarding the population with risk factors present, 42% started therapy with oxacillin and 24% with first-generation cephalosporins; however, due to therapeutic failure, the therapy was changed therapy to a higher-line antibiotic. In addition, 16% of the patients (112 patients) were infected with *MRSA* with risk factors for the development of bacterial resistance, such as the presence of devices and comorbidities. In this group of patients, second-line antibiotics (cefixime, cefotaxime, ceftazidime, linezolid, and clindamycin) were administered as first-line antibiotics, leading to therapeutic failure in the treatment of *MRSA* infections. The use of first-line and, consequently, second-line antibiotics is not always carried out; a study carried out in 15 Chinese hospitals in 2018 showed that healthcare workers had a greater tendency to administer second-line antibiotics in the face of an increase in antimicrobial resistance, fostered in previous years not only by an increased intake of antibiotics but also due to an increased use of antibiotics in the agro-food sector [44]. Therefore, we can deduce that in our facility, the setting of antimicrobial therapy respects the previous pattern, starting with the administration of a first-line antibiotic, but it is later modified with the administration of a second-line antibiotic in patients with *MRSA* infection and in many patients admitted to the intensive care unit.

In the intensive care unit, patients treated with oxacillin accounted for 19% (132 patients), whereas patients treated with cephalosporins accounted for 13% (17 patients). In the intensive care setting, a second-line antibiotic is preferred as an empirical therapy; in fact, 64% of patients were treated with aminoglycosides (399 patients), whereas 27% of patients were treated with fluoroquinolones (3 patients). Aminoglycosides are the class of antibiotics most used in patients admitted to the neonatal intensive care unit as an empirical antibiotic therapy, whereas fluoroquinolones are the class of antibiotics most commonly used in the pediatric intensive care unit, in combination with aminoglycosides or oxacillin, especially in oncology patients.

Regarding neonates in the intensive care unit, no randomized studies have demonstrated the absolute best choice of antibiotics. However, many authors agree that the combination of penicillin or semi-synthetic penicillin (ampicillin) together with an aminoglycoside is effective against microorganisms causing early-onset sepsis, and therefore can be considered the best empirical regimen. For the treatment of suspected late-onset sepsis, several authors agree that the best regimen is an anti-staphylococcal penicillin (oxacillin) together with an aminoglycoside; the choice of vancomycin should be limited to microbiologically proven cases of *MRSA* [45,46].

In all the groups examined, the factors mainly associated with the development of microbial resistance were being aged between 0 and 28 days, the presence of devices, and the presence of comorbidities. Risk factors for the development of resistance like those identified in our study were found in a cohort study performed between 2010 and 2014 in a group of pediatric patients aged between 30 days and 16 years, who were admitted to Hospital de Pediatria J.P. Garrahan due to infections caused by *S. aureus* were identified in blood cultures. The most frequently observed risk factor was the presence of a central venous catheter [47]. Interestingly, children with devices and admitted to the intensive care unit were more likely to have cephalosporin- and aminoglycoside-resistant *S. aureus* infections. Therefore, in these categories of patients, a high index of suspicion is required, and early use or switching to other classes of antibiotics should be considered in case of worsening during therapy or severe life-threatening infections. In our study, 79% of cases (956 patients) had *HA-MRSA* infection and 21% of cases (254 patients) had *CA-MRSA* infection. Patients with *HA-MRSA* infection had risk factors such as hospitalization in the previous year (29%), long-term hospitalization (25%), undergoing surgery (21%), presence of devices (70%), admission to the intensive care unit (42%), use of broad-spectrum antibiotics, and age under two years (34%). In fact, the highest prevalence of resistant *S. aureus* strains was found in patients with *HA-MRSA* infection. Patients with *CA-MRSA* infection, on the other hand, had the following criteria: diagnosis of the infection within 48 h after hospital admission, no previous *MRSA* infection, no previous surgery, no previous hospitalization, and no device.

Our study has certain limitations. First, the retrospective nature of this study may have been influenced by several methodological shortcomings. Surveillance studies should be conducted over longer periods of time and with a larger population. Previous antibiotic treatment, which this study was unable to assess due to its retrospective nature, should also be investigated in the future. Moreover, we analyzed the *S. aureus* resistance pattern to a small number of antibiotics that did not include other classes, such as macrolides, oxazolidinone, and tetracyclines. Another limitation is having analyzed the antibiotic resistance of *S. aureus* not only in the case of invasive infection but also in carriers, as our aim was to analyze patterns of antibiotic resistance and not an analysis of the disease severity of *S. aureus* infections. All these factors prompt us to conduct more in-depth research in the future. However, our cohort provides a comprehensive overview of the antibiotic susceptibility of *S. aureus* in children and antibiotic use in our area of interest and work. More studies should be conducted in the future to confirm our results.

## 5. Conclusions

In conclusion, our study showed that antibiotic resistance of *S. aureus* is progressively increasing in our environment, and patients with a history of ICU admission or the presence of devices are at higher risk. However, we also pointed out that adherence to antimicrobial practices is not always appropriate, highlighting the need for better education on this topic for pediatricians and neonatologists. Antimicrobial resistance is a major public health problem, and more attention should be paid to the pediatric population. Further epidemiological studies are needed to better understand the burden of antibiotic resistance in children and how exposure to antibiotics during childhood influences the development of resistance in the long term.

## Figures and Tables

**Table 1:** Baseline characteristics of participants.

1210 Participants	N° of Patients
Male	702 (58%)
Female	508 (42%)
0–28 days	484 (40%)
29–90 days	170 (14%)
>90 days	556 (46%)
Neonatal ICU	560 (46.2%)
Pediatric ICU	219 (18.1%)
Comorbidities	830 (68.6%)
Devices	814 (67.3%)

**Table 2:** Isolation site of a positive finding for *S. aureus* in relation to antibiotic resistance (PS: pharyngeal swab; BAL: bronchoalveolar liquid; CVC: central venous catheter).

Isolation Site:	Oxacillin	Cephalosporin	Aminoglycosides	Fluoroquinolones
PS (46%)	24.6%	15.2%	3.2%	2.3%
BAL (35%)	8.6%	16.4%	5.6%	4.4%
CVC (13%)	0.5%	0.4%	7.2%	4.9%
Urine (4%)	1.32%	0.6%	1.2%	0.91%
Other sites (2%)	0.6%	0.5%	0.57%	0.25%

**Table 3:** Oxacillin resistance (*MRSA*) analysis.

Oxacillin Resistance (*MRSA)*					
	YES		NO		
	*n*	%	*n*	%	*p*-Value
Age (days)					
0–28	235	72.53	89	27.47	<0.001
29–90	327	76.22	102	23.77	
>90	280	61.26	177	38.73	
Sex					
Male	425	60.54	277	39.45	0.811
Female	311	61.22	197	38.77	
ICU admission					
No	450	67.56	216	32.43	<0.001
Yes	219	40.25	325	59.74	
ICU type					
Neonatal ICU	144	59.01	100	40.98	0.997
Paediatric ICU	177	59.00	123	41.00	
Previous admissions					
No	582	69.70	253	30.29	<0.001
Yes	221	58.93	154	41.06	
Comorbidity					
No	416	57.37	309	42.62	<0.001
Yes	214	44.12	271	55.87	
Type of comorbidity					
Haematological	80	56.33	62	43.66	0.707
Gastrointestinal	60	42.25	55	38.73	
Infectious	63	57.27	47	42.72	
Kidney	39	48.14	42	51.85	
Other	19	51.35	18	48.64	
Device					
No	285	47.49	304	52.50	0.001
Yes	359	56.89	272	43.10	
Average days hospitalization	3.7		3.7		
Average emergency room access	1.09		1.09		

**Table 4:** Cephalosporin resistance analysis.

Cephalosporin Resistance					
	YES		NO		
	*n*	%	*n*	%	*p*-Value
Age (days)					
0–28	322	61.90	198	38.07	<0.001
29–90	210	48.38	224	51.61	
>90	147	57.42	109	42.57	
Sex					
Male	446	63.53	256	36.46	0.304
Female	308	60.62	200	39.37	
ICU admission					
No	384	56.38	297	43.61	<0.001
Yes	348	65.78	181	32.21	
ICU type					
Neonatal ICU	206	63.19	120	36.80	0.203
Paediatric ICU	117	57.63	86	42.36	
Previous admissions					
No	402	56.30	312	43.69	0.206
Yes	261	52.62	235	47.37	
Comorbidity					
No	467	60.72	302	39.27	0.027
Yes	239	54.19	202	45.80	
Type of comorbidity					
Haematological	53	54.08	45	45.91	0.620
Gastrointestinal	60	55.00	50	45.00	
Infectious	67	62.03	41	37.96	
Kidney	57	56.43	44	43.56	
Other	16	66.60	8	33.30	
Device					
No	300	50.08	299	49.91	<0.001
Yes	395	64.64	216	35.35	
Average days hospitalization	3.7		3.7		
Average emergency room access	1.09		1.09		

**Table 5:** Aminoglycosides resistance analysis.

Aminoglycosides Resistance					
	YES		NO		
	*n*	%	*n*	%	*p*-Value
Age (days)					
0–28	100	34.60	189	65.39	0.039
29–90	172	41.05	247	58.94	
>90	220	43.82	282	56.17	
Sex					
Male	294	41.88	408	58.11	0.249
Female	196	38.58	312	61.41	
ICU admission					
No	289	42.12	397	57.87	0.235
Yes	203	38.74	321	61.25	
ICU type					
Neonatal ICU	204	72.59	77	27.40	0.014
Paediatric ICU	152	62.55	91	37.44	
Previous admissions					
No	294	43.62	380	56.37	0.907
Yes	232	43.28	304	56.71	
Comorbidity					
No	266	44.93	326	55.06	0.002
Yes	223	36.08	395	63.91	
Type of comorbidity					
Haematological	34	29.56	81	70.43	0.087
Gastrointestinal	49	35.25	90	64.74	
Infectious	31	20.66	119	79.33	
Kidney	36	26.08	102	73.91	
Other	20	26.31	56	73.68	
Device					
No	357	56.04	280	43.95	0.578
Yes	312	54.45	261	45.54	
Average days hospitalization	3.7		3.7		
Average emergency room access	1.09		1.09		

**Table 6:** Fluoroquinolones resistance analysis.

Fluoroquinolones Resistance					
	YES		NO		
	*n*	%	*n*	%	*p*-Value
Age (days)					
0–28	220	36.91	376	63.08	0.870
29–90	132	37.07	224	62.92	
>90	100	38.75	158	61.24	
Sex					
Male	289	41.16	413	58.23	0.623
Female	202	39.76	306	60.23	
ICU admission					
No	273	39.85	412	60.14	0.491
Yes	199	37.90	326	62.02	
ICU type					
Neonatal ICU	130	59.09	90	40.90	0.662
Paediatric ICU	186	60.98	119	39.01	
Previous admissions					
No	217	30.13	503	69.86	0.006
Yes	185	37.75	305	62.24	
Comorbidity					
No	222	32.31	465	67.68	0.364
Yes	182	34.79	341	65.20	
Type of comorbidity					
Haematological	30	25.42	88	74.57	0.017
Gastrointestinal	39	33.62	77	66.37	
Infectious	49	44.95	60	55.04	
Kidney	32	29.09	78	70.90	
Other	19	27.14	51	72.85	
Device					
No	271	34.08	524	65.91	<0.001
Yes	265	63.85	150	36.14	
Average days hospitalization	3.7		3.7		
Average emergency room access	1.09		1.09		

## Data Availability

Data are available upon reasonable request to the corresponding author.
